# Orthodontic Molar Brackets: The Effect of Three Different Base Designs on Shear Bond Strength

**Published:** 2011-03

**Authors:** Athol P. Hudson, Sias R. Grobler, Angela M. P. Harris

**Affiliations:** 1 *Department of Orthodontics, University of the Western Cape, Cape Town, South Africa;*; 2 *Oral and Dental Research Institute, University of the Western Cape, Cape Town, South Africa*

**Keywords:** base design, molars, shear bond strength, stainless steel brackets

## Abstract

The purpose of the study was to assess the relative base designs of three different maxillary molar stainless steel brackets with reference to the shear bond strength of three different adhesive resins. The molar brackets used were Victory series (3M Unitek), Upper Molar (GAC) and Optimesh XRT (Ormco). The adhesives used were Transbond XT (3M Unitek), Enlight (Ormco) and Sure Ortho Light Bond (Sure Orthodontics). The human enamel specimens (144) were randomly divided into nine groups and each group (n=16) was allocated to a bracket/adhesive combination. The contact surface of each of the bracket bases was measured three dimensionally using a reflex microscope. The base designs were also subjected to further microscopic investigations. The brackets were bonded to the enamel, temperature cycled and the shear bond strength was measured. The size and design of each of the brackets was different. The base size, surface treatment, mesh strand diameter and aperture size of the bracket base mesh have a significant effect on the shear bond strength at the bracket/adhesive interface. The shear bond strengths of all three Ormco bracket/adhesive resin combinations (5.8-6.8 MPa) were significantly lower (*p*<0.05; Kruskal-Wallis) than the other six bracket/adhesive combinations (9.4-12.1 MPa). The different adhesive types (3 types) could not be mainly responsible for the low shear bond values found for the Ormco bracket. The 3M Unitek combination of the Victory series bracket and Transbond XT adhesive proved to have a high shear bond strength without enamel damage.

## INTRODUCTION

Enamel etching is a clinically accepted procedure even though there is enamel decalcification and loss (1, 2). Modern fixed appliances are routinely bonded from second molar to second molar and are associated with a great deal of success (3). It has however been shown that molar bonding is associated with survival times almost half that of cemented bands on first molars (4). Environmental forces in the molar region are considered to play an important role in the success or failure of molar bonding (4). Forces of up to 360 Newtons have been registered in the molar region in young adolescents (5). Other key factors in molar bonding are operator accessibility, moisture control, the depth of the bite and patient dietary co-operation (4). Normal orthodontic forces applied to the brackets are estimated to produce stresses in the region of 3 to 7.8 MPa, while it is suggested that for an adhesive system to have a clinically acceptable performance, the *‘in vitro’* bond strength should be between 6 and 8 MPa (6, 7).

The variables associated with shear bond strength are amongst others the size, design and the surface treatment of the adhesive contact surface of the bracket bases (8, 9). In the attempt to improve bond strength the bracket adhesive pad has been a focus of development and base sizes have been reduced considerably in recent years (10). The demands of an aesthetically conscious society, improved adhesive systems and the refinement of bracket base design have enabled manufacturers to decrease the size of the bracket bases, without sacrificing bond strength (4, 11, 12). Manufacturers claim their own unique ‘in house’ adhesive surface design, trademarks and/or patents, but at the same time provide very little information regarding the dimensions of these bases (13). The size of the bracket base is important because of the following considerations: oral hygiene, bond strength and aesthetics (3, 8, 13-15).

There are various bracket base designs all in an attempt to optimise the mechanical bond between the bracket and the adhesive. The design of the bracket base adhesive pad has been found to be a significant factor in mean shear bond strength (8). Seventy five percent of brackets with a simple foil mesh base undergo bond failure at the bracket adhesive interface (16). Presently most stainless steel orthodontic brackets have a fine mesh design (12) on the adhesive surface of the bracket base (17, 18).

It has been reported that mesh based brackets with larger mesh spaces (apertures) provide a greater shear bond strength than do bases with smaller mesh apertures (10). The number of openings per unit of area of the bracket base is determined by the wire diameter and the mesh spacing. For resin to penetrate the base effectively air needs to be able to escape and this is determined by the free volume between the mesh and the bracket base (12, 19). As far as the mesh design is concerned, Matasa (13) claimed that the mesh number and the wire diameter of the mesh are the most important influencing factors. The two areas in which improvements have taken place are in the design of the mesh as well as the use of bond enhancing metal surface treatments applied to the mesh (10, 19). The various types of treatment applied to bracket bases have entailed micro-etching, sandblasting, polymer coating or a spray with fine particles of molten metal (13). The current trend is for a less dense mesh to be used so as to ensure a larger aperture or open area in the base (17).

It has been demonstrated that retentive surface enlargement improves adhesion but also increases the risk of fracture at the bracket/adhesive interface because of surface variability (11). This substantiates the finding by MacColl (19) that shear bond strength is independent of the base size once the surface area of the bracket exceeds 7 mm^2^.

The purpose of the study was to assess the relative base designs of three different maxillary molar stainless steel brackets with reference to the shear bond strength of three different adhesive resins.

## MATERIALS AND METHODS

### Technical information

Maxillary molar stainless steel orthodontic brackets from each of three different manufacturers were obtained. These were:

- Optimesh XRT (Ormco, Orange, CA92867. U.S.A.) Lot: 06D238D
- Victory Series (3M Unitek, Monrovia, CA 91016. U.S.A.) Lot 998186100
- Bondable molar attachment (GAC, Bohemia, NY 11716. USA.) Lot B375

Each of the abovementioned brackets had their 3 dimensional contact surface area measured making use of a reflex microscope (Prior S2000 Reflex Microscope, 9 Whitehall Park, London. N19. No 001). The readings were taken in a mesiodistal direction parallel to the mesh strands from the occlusal to the gingival aspect of the bracket base. The readings were taken to include the undulations of the mesiodistal mesh strands in 3 vertical planes. The floor of each aperture was read in 9 locations (3 measurements in three different horizontal planes). Each vertical mesh strand when encountered had its profile measured in 3 different horizontal locations. The largest of the bases was measured by performing approximately 3000 readings. Each of the bracket types had 2 different specimens measured and the average was taken.

The mesh wire diameter of each bracket was measured in microns using the Zwick/Roell ZHV microhardness tester (Indentec hardness testing machines limited, West Midlands, DY9 8HX). The aperture dimensions were also measured using the same apparatus and the open space or aperture area was calculated. Each bracket type was measured randomly in five locations for both mesh strand thickness and for the size of the open spaces (apertures). Each of the bracket bases was sectioned in order to measure the average depth of the apertures of each of the brackets.

Microscopic images of the adhesive surfaces of the 3 different brackets were captured. This was done by laying the same square millimetre micrometer eyepiece (Olympus Corporation, Tokyo. Japan.) on the mesh surface of each of the brackets. The micrometer was placed upside down in order to have lines of the grid as close as possible to the mesh of the base. The bracket was photographed at 40 and 140 times magnification under the same lighting conditions. The number of apertures in the mesh of each of the bases were calculated and expressed as apertures per square millimetre.

These microscopic investigations were performed in order to make comparisons between the 3 base designs.

The three adhesive resins used to bond each of the brackets were:

- Enlight (Ormco, Orange, CA92867. U.S.A.)
- Transbond XT (3M Unitek, Monrovia, CA 91016. U.S.A.)
- Sure Ortho Light Bond (Sure Orthodontics, Geneva. Switzerland).

One hundred and forty four (144) upper extracted human molar enamel specimens were selected. Teeth with caries affecting or undermining the buccal enamel were excluded, as were all teeth exhibiting fluorosis or enamel damage as result of the extraction process.

The selected teeth were prepared for bonding by sectioning them in such a way as to remove the roots. This was done by means of a water cooled high speed turbine handpiece. The sectioned crowns were stored in water at four degrees centigrade with a few crystals of thymol added (as an anti-bacterial agent). The teeth were randomly divided into three groups, one group of forty eight specimens for each of the bracket groups. Each group of these assigned teeth was then divided into three subgroups of sixteen teeth each and bottled (9 bottles) separately. Each of these bottles was labeled with the assigned bracket/adhesive resin combination. This was done with a view to ensure that sixteen brackets of each manufacturer would be bonded with each of the three resin adhesive agents (16 × 3 × 3 combinations). Each enamel specimen was then checked by a single operator in order to ensure a close fit between the bracket base and the buccal surface of any of the assigned enamel specimens. This was done by placing an example of its assigned bracket with its base positioned in the prescribed position on the buccal enamel. If there was any doubt regarding the closeness of the ‘fit’ of the base to the buccal tooth surface, the tooth was excluded from any further testing and another specimen was assessed and used if found to be suitable. This was done in an attempt to minimise the variation of the thickness of the adhesive layer as much as possible.

All the enamel specimens were gently polished for 10 seconds with an oil free, fluoride free pumice solution to clean the enamel, thus simulating the removal of the pellicle as in the clinical situation.

All the brackets to be bonded with the same bonding agent were bonded in one session by a single operator. Each of the three adhesive resins was used in accordance with the manufacturer’s instructions. The bracket was positioned on the buccal surface of each tooth, by means of bracket tweezers, and then a force of four hundred grams (20) was applied by means of a Dontrix gauge (American Orthodontics, Sheboygan, Wisconsin, WI 53081. U.S.A.). Prior to light curing the excess adhesive agent was removed from around the base of the bracket with a sharp probe.

The adhesive resin on each bonded tooth was light cured in three steps for ten seconds at the mesial side, then for 10 seconds at the occlusal side and finally for 10 seconds at the distal side of the bracket with the exit portal of the light curing unit as close as possible to the bracket. A standard tungsten quartz halogen curing light (Optilux 501, Demetron Research Corporation) was used to cure the bonding agents. A light intensity range of between 440 and 480 mW/cm^2^ was used. The intensity of the curing light was checked after every 8 exposures with a Dentsply light intensity meter (Cure Rite Meter, Dentsply, Caulk) to ensure this consistent intensity. Each bonded specimen was placed back into the water/thymol solution in its designated bottle and stored for twenty four hours at room temperature and then exposed to a temperature cycling procedure. This entailed each specimen being exposed to 500 cycles of heat and cold. The specimens were exposed to a temperature high of 55°C as opposed to a low of 5°C, with a dwell time of 30 seconds.

Following the temperature cycling the enamel specimens were stored in their respective adhesive/bracket combination groups. The bonded enamel specimens were then embedded in plastic cups with cold curing acrylic resin. The specimens were positioned by means of a jig in such a way that the entire buccal enamel surface projected from the embedding material and the plastic tube with the bracket/enamel interface positioned at ninety degrees to the long axis of the plastic tube. The specimens were kept in water between all treatments.

The specimens were clamped to the base of the Zwick Universal testing machine (Materialprufung, 1446, Germany). A shear load was applied in an occluso-gingival direction to the attachment, with the debonding force parallel to the bracket/adhesive interface by means of a knife-edged rod at a crosshead speed of 0.5 mm per minute. Care was taken to ensure that the debonding force was consistently applied at the junction between the base of the bracket and the buccal tube on all of the specimens. Shear bond strengths were registered in Newtons to be converted and expressed in mega pascals (MPa) using the contact surface area as reported in Table 1. The results were then subjected to the Kruskall-Wallis as well as the Tukey-Kramer multiple comparison tests. An adhesive resin index was performed on teeth after de-bonding.

## RESULTS

The average bracket base contact surface area of the 3M, GAC and Ormco brackets as measured with the reflex microscope appear in Table 1.

Table 2 shows the average base mesh dimensions of the 3M, GAC and Ormco brackets.

The pivot table (Table 3) shows average shear bond strength values of the 3M bracket/adhesive combinations ranged from 9.8-11.8, of the GAC from 9.2 to 11.7 MPa and for the Ormco combinations 5.8-7.6.

Table 4 gives the adhesive remnant index per bracket/adhesive combination on an ARI scale of 0-4, based on the index used by Kirovski and Madzarova (21).

**Figure 1:**
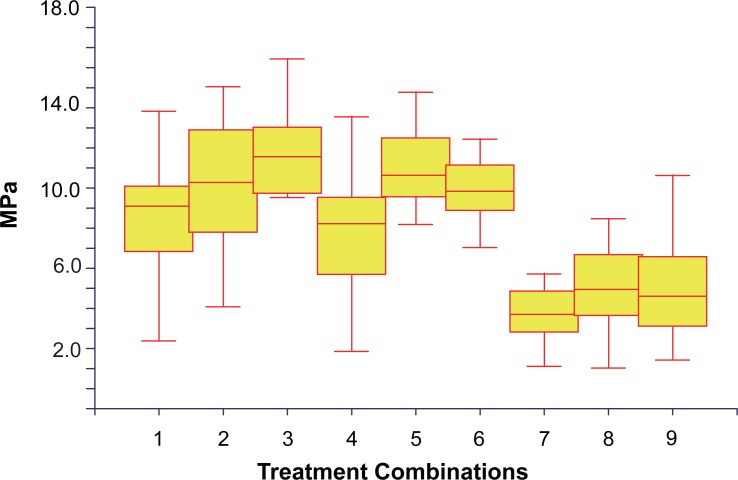
A box plot showing the shear bond strengths in MPa of each treatment combination. Treatment combinations: 1, 3M bracket/Enlight adhesive; 2, 3M bracket/Sure Ortho Light Bond adhesive; 3, 3M bracket/Transbond XT adhesive; 4, GAC bracket/Enlight adhesive; 5, GAC bracket/Sure Ortho Light Bond adhesive; 6, GAC bracket/Transbond XT adhesive; 7, Ormco bracket/Enlight adhesive; 8, Ormco bracket/Sure Ortho Light Bond adhesive; 9, Ormco bracket/Transbond XT adhesive.

Figure 1 shows the Box and Whisker plot of shear bond strengths (MPa) of the mentioned 9 different combinations. The intermediary box represents the range of 50% of the shear bond values, the top line of the box shows the value for the first quartile, the bottom value the 3^rd^ quartile and the line within the box represents the median value. The region between the 1^st^ and 3^rd^ quartile values is known as the interquartile range.

**Figure 2:**
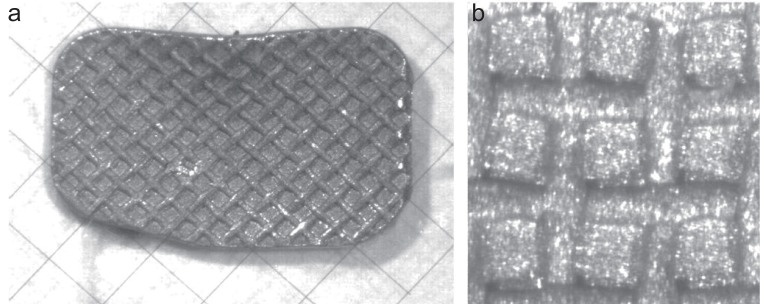
The 3M Victory series bracket in the “as received” condition photographed at 40× and 140× magnification. a, At 40× magnification a glass millimeter grid was placed on the bracket base and photographed; b, At 140× magnification an area of approximately 1 mm^2^ is shown.

**Figure 3:**
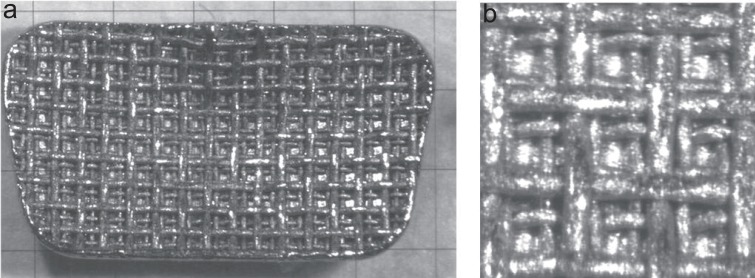
The GAC molar bracket in the “as received” condition photographed at 40× and 140× magnification. a, At 40× magnification a glass millimeter grid was placed on the bracket base and photographed; b, At 140× magnification an area of approximately 1 mm^2^ is shown.

**Figure 4:**
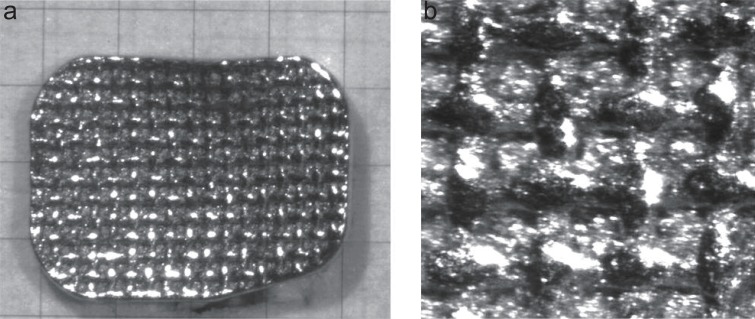
The Ormco bracket in the “as received” condition photographed at 40× and 140× magnification. a, At 40× magnification a glass millimeter grid was placed on the bracket base and photographed; b, At 140× magnification an area of approximately 1 mm^2^ is shown.

Figures 2, 3 and 4 are microscopic images of the adhesive surfaces of the 3M, GAC and Ormco brackets, respectively.

The shear bond strengths associated with all 3 Ormco bracket/adhesive combinations were found to be significantly lower (*p*<0.05; Kruskal-Wallis) than all the other combinations (Figure 1, Table 3).

**Table 1:** The average bracket base size as measured with a reflex microscope

Bracket make	Contact surface area

GAC	26.20mm^2^
3M	25.05mm^2^
Ormco	20.90mm^2^

**Table 2:** Comparative table of the average base mesh dimensions of each of the brackets used in this study

	Bracket bases		
	3M	GAC	Ormco

**Aperture area (μm^2^) (length × width)**	42640	49500	19600
**Average thickness of the mesh strands (μm)**	115.5	113.5	126.5
**Average depth of the apertures (μm)**	126.9	156.7	124.9
**Average aperture volume (μl)**	5.43	7.72	2.47

**Table 3:** A pivot table of shear bond strength expressed in MPa for the contact surface area (average, standard deviation, minimum and maximum)

Adhesive	3M brackets			GAC brackets			Ormco brackets		
	Enl	SOLB	Tb	Enl	SOLB	Tb	Enl	SOLB	Tb

Count	16	16	15^a^	16	16	16	16	16	16
Average	9.8	10.9	11.8	9.2	11.7	10.4	5.8	7.6	7.0
Standard deviation	2.2	2.5	2.8	2.6	1.6	2.1	1	3.5	2.6
Minimum	4.7	6.1	5.5	4.3	9.3	4	3.7	3.6	3.9
Maximum	13.9	14.8	16.0	13.6	14.6	12.7	7.4	17.2	14.1

a One specimen lost during testing; Enl, Enlight adhesive resin; SOLB, Sure Ortho Light Bond; Tb, Transbond XT.

**Table 4:** The adhesive remnant index per bracket/adhesive combination

		ARI Score per 16 teeth				

Bracket	adhesive	0	1	2	3	4

3M-Unitek	Transbond XT		10^a^	4	1	
Ormco	Transbond XT			8	8	
GAC	Transbond XT	3	8		3	2
3M-Unitek	Enlight		5	3	7	1
Ormco	Enlight			5	11	
GAC	Enlight	1		3	5	7
3M-Unitek	Sure Ortho Light Bond			3	9	4
Ormco	Sure Ortho Light Bond		1	5	10	
GAC	Sure Ortho Light Bond		1		6	9

**The description of each category of the adhesive remnant index**						

**ARI**	**Description**					

0	0% of the bonding agent remaining on the enamel specimen surface.						
1	Less than 50% of the bonding agent remaining on the enamel specimen surface.						
2	50% or more of the bonding agent remaining on the enamel specimen surface.						
3	100% of bonded enamel covered by a layer of bonding agent						
4	Enamel damage as a result of debonding.						

a One specimen was lost during testing.

## DISCUSSION

The adhesive surfaces of each of the three tested brackets have mesh bases, each with a different design (Figures 2-4). Upon microscopic investigation, the differences in the design of the adhesive surfaces of the bracket bases were evident. The 3M brackets (Figure 2) displayed a single mesh design with the mesh criss-crossing the base diagonally from corner to corner. The entire base had an even mat finish. The GAC brackets (Figure 3) displayed a double mesh structure on the base. The entire base also had an even mat finish. The Ormco bracket (Figure 4) surface displayed a single mesh layer. The surface of the mesh appeared to be shiny and smooth whilst the ‘roof’ of the apertures had a rough and irregular surface.

The Ormco bracket base showed 4 openings per lineal millimetre or 16 openings per square millimetre (Figure 4). While, both the 3M and GAC bracket bases each showed 3 openings per lineal millimetre or 9 openings per square millimetre (Figures 2 and 3).

The shear bond strengths of all three of the Ormco bracket/adhesive resin combinations were shown to be significantly different (*p*<0.05) from any of the other 6 bracket/adhesive combinations in this study (Figure 1). The shear bond strengths associated with the Ormco brackets were also the lowest. Within each bracket group all 3 of the adhesives were associated with shear bond strengths of a similar magnitude (Figure 1). Thus, the different adhesive types (3 types) could not be mainly responsible for these low values found for the Ormco bracket but rather the other features discussed below.

It was interesting to note that in association with all three bracket groups Enlight adhesive resin (Ormco) was consistently associated with a lower shear bond strength (Figure 1). Ormco does not disclose the particle size in Enlight while 3M claims that Transbond XT contains submicron silica particles (22) and Sure Ortho Light Bond is quoted as containing nano silica (personal communication with Barry Zalsman barry@bjmlabs.com).

Some authors claim that a reduced bracket base contact surface size does not significantly affect the shear bond strength (4, 12, 13). It has also been stated that a smaller bracket base size is an important variable that could affect bond strength (8). However, none of the abovementioned studies refer to molar bonding. The results of this study showed that the Ormco bracket base size (20.90 mm^2^) is roughly twenty percent less than that of the GAC bracket base and roughly fifteen percent smaller than the 3M bracket base (Table 1). But the base size reduction of the Ormco bracket is related to shear bond strength reductions of up to forty percent (Table 3). In light of this it must be assumed that there must also be other variables associated with the Ormco bracket other than size that fulfil important roles in determining shear bond strength values. Other variables to consider are: The contact surface design, any treatment applied to the base of the bracket or the adhesive system used. A mesh base consists of multiple fine wire strands woven at ninety degrees to one another. Therefore the wire diameter and the size of the apertures between the mesh strands are variables that may affect shear bond strength. The Ormco bracket base average aperture area was less than half the size of the average aperture areas (19600 μm^2^) of either the GAC or the 3M bracket contact surfaces (Table 2). Furthermore, the Ormco brackets have the largest mesh strand diameter (126.5 μm), the smallest mesh apertures and aperture area (19600 μm^2^), the smallest aperture volume and more apertures per lineal millimetre (Figure 4) when compared to the bases of the either the 3M or GAC brackets used in this study (Figures 2, 3). The volume of the aperture is thought to play a critical role in allowing the air to be displaced from the contact surface as well as allowing more resin particle penetration into the apertures (13). The shear bond strengths of the 3M bracket combinations did not differ significantly from that of the GAC combinations (Figure 1). The base dimensions of the 3M and GAC brackets do not differ significantly as far as the aperture area, the diameter of the mesh strands, the depth of the apertures and the aperture volume (Table 2).

The other difference which might have an influence is the contact surface treatment. The Ormco bracket contact surfaces are treated with an Optimesh^®^ XRT coating. The Ormco product catalogue claims that this treatment increases the surface area of the contact surface by upto 35% (23). The 3M bracket has a mat appearance as a result of micro-etching and the GAC bracket has a similar appearance due to sandblasting. This roughening of the surface also increases the microscopic contact surface area of the mesh. By comparing Figures 2, 3 and 4 which were exposed to similar lighting conditions the Ormco bracket appears to be more reflective thus suggesting a smoother surface.

Retief (24) demonstrated *in vitro* enamel fracture at 9.7 MPa. Enamel fracture on the debonding of metal brackets is an occurrence that is not commonly associated with the clinical situation (4, 25, 26). It is noted in the literature that laboratory testing procedures provide higher bond strengths than those obtained in the clinical situation. This is thought to be a result of the possibility of moisture contamination, access and inter-operator differences in the clinical arena (4, 27).

The adhesive remnant index (ARI) analysis (Table 4) showed that none of the Ormco bracket/adhesive combinations caused enamel fracture on debonding and that almost all the debonding occurred at the bracket/adhesive interface. The Ormco brackets showed a highly statistically significant negative correlation (Spearman Rank correlation *p*<0.001) between the incidence of debonding at the adhesive enamel interface and the adhesive remnant index. The 3M bracket/adhesive combinations showed visible enamel damage in 5 instances after debonding. The GAC bracket/adhesive combinations were associated with 18 incidents of visible enamel damage and it was the only bracket/adhesive combination to remove all the adhesive resin from the enamel surface in 4 instances. The incidence of enamel fracture with GAC brackets was shown to be significant (*p*<0.05). Chi-square of 62.1438. The apparent stronger bond at the bracket/adhesive interface on the debonding of the GAC bracket may be associated with the larger average aperture volume size. GAC claim (GAC US patent 4889485) the double mesh design enhances the bond at the bracket/adhesive interface as well as it serves to reduce the amount of residual adhesive left on the enamel. The bracket construction is such that the mesh material becomes thicker, the apertures wider and the surfaces rougher toward the adhesive/bracket interface. Mesh design has been found to have an effect on stress distribution at debonding mainly by influencing the flexibility of the base of the bracket (28). Double mesh bases have been found to show less stress in the superficial mesh as opposed to the deeper mesh layer thus allowing increased flexibility of the base, when compared to single mesh designs. Wire diameter and mesh spacing of the single mesh brackets affect the size and location of the stresses both adhesively and cohesively (28).

Bishara *et al* concluded that single and double mesh bases display similar bond strength and bracket failure modes (29). The results of this study however do not concur with those findings. The GAC bracket displayed a significant incidence of enamel damage. The GAC and 3M brackets did however display similar shear bond strengths. There were significant differences between the GAC and Ormco brackets, as far as shear bond strength and bracket failure mode were concerned. Thus confirming that bracket base size and in particular mesh design are crucial elements of bond strength. In agreement to our study, Chapman (30) also found that two different base designs (single mesh and double mesh), three different adhesives as well as different combinations of these adhesives and brackets all had an influence on shear bond strengths.

The 3M Unitek combination of the Victory series bracket and Transbond XT adhesive proved unique in these tests. The 3M bracket/Transbond XT combination was the only bracket/adhesive combination other than the Ormco bracket/ adhesive combinations not to display any visible enamel damage.

## CONCLUSION

- The size and design of the bracket adhesive surface do play a significant role in bond strength of molar orthodontic brackets.
- The Ormco bracket has the largest mesh strand diameter, the smallest mesh apertures and aperture area, the smallest aperture volume and more apertures per lineal millimetre.
- The 3M Unitek combination of the Victory series bracket and Transbond XT adhesive proved to have a high shear bond strength without enamel damage.

## References

[1] Hosein I, Sherriff M, Ireland AJ (2004). Enamel loss during bonding, debonding, and cleanup with use of a self- etching primer. Am. J. Orthod Dentofacial Orthop.

[2] Flores AR, Saez GE, Barcelo F (1999). Metallic bracket to enamel bonding with a photopolymerizable resin-reinforced glass ionomer. Am. J. Orthod Dentofacial Orthop.

[3] Tsibel G, Kuftinec MM (2004). A bonded transpalatal arch. J. Clin. Orthod.

[4] Banks P, Macfarlane TV (2007). Bonded versus banded first molar attachments: a randomized controlled clinical trial. J. Orthod.

[5] Sonnesen L, Bakke M (2005). Molar bite force in relation to occlusion, craniofacial dimensions and head posture in pre-orthodontic children. Eur. J. Orthod.

[6] Reynolds IR (1975). A review of direct orthodontic bonding. Br. J. Orthod.

[7] Clark SA, Gordon PH, McCabe JF (2003). An ex vivo investigation to compare orthodontic bonding using a 4-META based adhesive or a composite adhesive to acid etched and sandblasted enamel. J. Orthod.

[8] Sharma-Sayal SK, Rossouw PE, Kulkarni GV, Titley KC (2003). The influence of orthodontic bracket base design on shear bond strength. Am. J. Orthod. Dentofacial Orthop.

[9] Bishara SE, VonWald L, Olsen ME, Laffoon JF (1999). Effect of Time on the Shear Bond Strength of Glass Ionomer and Composite Orthodontic Adhesives. Am. J. Orthod Dentofacial Orthop.

[10] Matasa CG (2003b). In Search of a Better Bond: State of the Art. Orthod Mat Insider.

[11] Cozza P, Martucci L, De Toffol L, Penco SI (2006). Shear bond strength of metal brackets on enamel. Angle Orthod.

[12] Cucu M, Driessen CH, Ferreira PD (2002). The influence of orthodontic bracket base diameter and mesh size on bond strength. S. Afr. Dent. J.

[13] Matasa CG (2003a). Do Adhesives and Sealants Really Seal the Brackets’ Pad? II. Surface Tension. Orthod Mat Insider.

[14] Sperber RL, Watson PA, Rossouw PE, Sectakof PA (1999). Adhesion of orthodontic attachments to dental amalgam: *in vitro* study. Am. J. Orthod Dentofacial Orthop.

[15] Millett DT, McCabe J (1996). Orthodontic bonding with glass ionomer cement; a review. Eur. J. Orthod.

[16] Sorel O, El Alam R, Chagneau F, Cathelineau G (2002). Comparison of bond strength between simple foil mesh and laser-structured base retention brackets. Am. J. Orthod Dentofacial Orthop.

[17] Wang WN, Li CH, Chou TH (2004). Bond strength of various bracket base designs. Am. J. Orthod Dentofacial Orthop.

[18] Maijer R, Smith DC (1981). Variables influencing the bond strength ofmetal orthodontic bracket bases. Am. J. Orthod.

[19] MacColl GA, Rossouw PE, Titley KC, Yamin C (1998). The relationship between bond strength and orthodontic bracket base surface area with conventional and microetched foil-mesh bases. Am. J. Orthod Dentofacial Orthop.

[20] Kirovski I, Madzarova S (2000). Tensile bond strength of a light cured cement when used for bracket bonding under different conditions: an *in vitro* study. Europ. J. Orthod.

[21] Grubisa HIS, Heo G, Raboud D (2004). An evaluation and comparison of orthodontic bracket bond strengths achieved with self-etching primer. Am. J. Orthod Dentofacial Orthop.

[22] Bishara SE, Ajlouni R, Laffoon JF, Warren J (2002). Effects of modifying the adhesive composition on the bond strength of orthodontic brackets. Angle Orthod.

[23] Ormco Product catalogue.

[24] Retief DH (1974). Failure at the dental adhesive etched enamel interface. J. Oral Rehabil.

[25] Summers A, Kao E, Gilmore J (2004). Comparison of bond strength between a conventional resin adhesive and a resin modified glass ionomer adhesive: An *in vitro* and *in vivo* study. Am. J. Orthod Dentofacial Orthop.

[26] Pickett KL, Sadowsky PL, Jacobson A, Lacefield W (2001). Orthodontic *in vivo* bond strength: Comparison with *in vitro* results. Angle Orthod.

[27] Rix D, Foley TF, Mamandras A (2001). Comparison of bond strength of three adhesives: Composite resin, hybrid GIC, and glass filled GIC. Am. J. Orthod Dentofacial Orthop.

[28] Knox J, Kralj B, Hubsch P (2001). An evaluation of the quality of orthodontic attachment offered by single and double-mesh brackets using the finite element method of stress analysis. Angle Orthod.

[29] Bishara SE, Soliman MM, Oonsombat C (2004). The effect of variation in mesh base design on shear bond strength of orthodontic brackets. Angle Orthod.

[30] Chapman JL, Corell MN, Armbruster PC, Du JX (2009). Shear bond strength of molar tubes bonded with different adhesives. Aust Orthod.

